# Mixoma Atrial Recorrente em Paciente com Complexo de Carney. Relato de Caso e Revisão de Literatura

**DOI:** 10.36660/abc.20190405

**Published:** 2020-05-11

**Authors:** Laura A. Cervantes-Molina, David Ramírez-Cedillo, Italo D. Masini-Aguilera, Jaime G. López-Taylor, Michel Machuca-Hernández, Dulman O. Pineda-De Paz

**Affiliations:** 1 Hospital Civil de Guadalajara Unidad Hospitalaria Fray Antonio Alcalde GuadalajaraJalisco México Hospital Civil de Guadalajara Unidad Hospitalaria Fray Antonio Alcalde - Cardiovascular Surgery, Guadalajara, Jalisco - México

**Keywords:** Complexo de Carney/complicações, Mixoma Atrial, Loci Gênicos, Morte Súbita Cardíaca, PRKAR1A

## Introdução

Tumores cardíacos primários são incomuns. Apresentam incidência entre 0,0017% e 0,28%, correspondendo a 17 e 2.800 tumores cardíacos primários a cada 1 milhão de autópsias.^1^ O mixoma cardíaco (MC) é uma neoplasia benigna e representa a mais comum entre os tumores cardíacos primários em adultos.^2^

O MC apresenta uma incidência anual de 0,5–0,7 de casos de ressecção cirúrgica a cada um milhão; com a maioria dos casos apresentando aparência esporádica e, menos de 10%, apresentando padrão de hereditariedade familiar.^3^

O átrio esquerdo (AE) é o local de origem mais comum (75–80%), seguido do átrio direito. MC múltiplos representam 5% de todos os CMs e apenas 50% deles têm origem bilateral.^4^

O MC apresenta diversos tipos de manifestações, principalmente sintomas obstrutivos, podendo produzir embolias, sendo o pior dos cenários.^1^

Existem 2 tipos de MC: 1. O tipo simples, o mais comum, representando 95% de todos os MCs. Sua maior prevalência é aos 56 anos, com risco de 1 a 3% de desenvolver um segundo mixoma. 2. Formas familiares autossômicas dominantes, como o Complexo de Carney (CNC).^2^

Esses tipos de CM apresentam uma distribuição anatômica atípica que é diferente do AE.^5^ Eles aparecem aos 25 anos de idade, em média, e tendem a ser múltiplos em 45% dos casos, com uma taxa de recidiva de 15% a 22%.^1 , 4 , 6^

## Relato de caso

**Figura 1:**
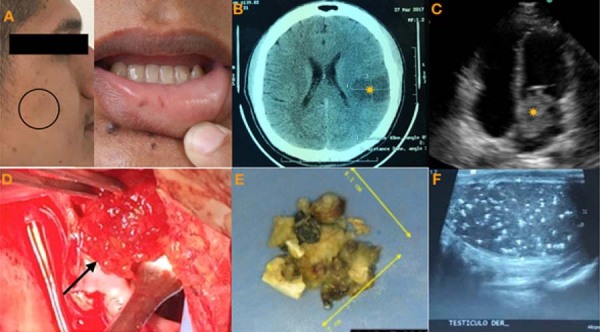
- Painel A. Imagem do paciente mostrando nevos acinzentados (seta) e manchas café com leite (círculo). Painel B. Tomografia computadorizada (TC) axial do crânio mostrando imagem hipodensa intra-axial na região parieto-temporal esquerda de 40 * 24 mm (asterisco) com edema circundante leve/moderado. Painel C. Corte apical transtorácico de quatro câmaras mostrando grande massa atrial esquerda (asterisco) e painel D mostrando sua correlação com a vista cirúrgica (seta). Painel E. Vista macroscópica do mixoma do átrio esquerdo. Painel F. Ultrassom testicular direito mostra múltiplas calcificações.

Relatamos o caso de um paciente do sexo masculino, com 22 anos de idade, histórico de ressecção de MC no átrio direito aos 12 anos de idade e ressecção de mixoma cutâneo abdominal aos 20 anos de idade; ele foi levado ao pronto-socorro devido a parestesia generalizada de início súbito associado a hemiparesia fascio-corporal direita e afasia motora. Ao exame físico, aparência acromegálica, pescoço curto, lentiginose com nevos acinzentados no lábio inferior e múltiplas manchas café com leite na face ( Figura 1-A ).

A tomografia computadorizada axial do crânio mostrou imagem hipodensa intra-axial na região parietal-temporal esquerda com 40 x 24 mm de diâmetro ( Figura 1-B ). Em seguida, foi realizada ecocardiografia, que mostrou FEVE normal (64%) e dilatação leve do AE. Massa móvel do AE (5,4 cm x 2,8 cm de diâmetro) de aparência homogênea e contornos regulares aderidos ao septo interatrial com prolapso para o ventrículo esquerdo sem gradiente de estenose ou sinais de regurgitação. ( Figura 1-C )

Suspeitou-se de CNC, devido a um perfil hormonal anormal [hipertireoidismo secundário e hipercortisolismo]. ( Tabela 1 )

**Tabela 1:** – Perfil endócrino

IGF-1	922 ng/ml (z. Escore: +4,8)
ACTH	22,6 µmol/l
PRL	3,42 ng/dl
Cortisol	33,01 mcg/dl
TSH	7,26 mcU/ml
T4l	2,34

Exames adicionais foram realizados: ressonância magnética cerebral, que mostrou, na sequência axial T1, área hipointensa de 7 mm de diâmetro, correspondendo a microadenoma hipofisário e ultrassonografia testicular, que revelou microlitíase bilateral ( Figura 1-F ).

Conforme recomendado nas diretrizes internacionais, o paciente foi encaminhado à cirurgia cardíaca para ressecção do tumor e ressecção concomitante do septo atrial, por ser absolutamente obrigatório durante a ressecção do MC em casos de CNC para evitar a recidiva do mixoma atrial. Foi encontrada massa de AE de consistência macia e friável, compatível com MC de 5,5 cm x 3 cm ( Figura 1-D e E ). A análise histológica, novamente, confirmou o mixoma atrial.

O paciente apresentou desfecho pós-operatório favorável, sem nenhuma complicação, além de melhora progressiva dos sintomas neurológicos. O diagnóstico de CNC foi estabelecido principalmente devido a múltiplos distúrbios cutâneos, MC “recorrente” e “bilateral”, histórico de mixoma extracardíaco, além de distúrbios endócrinos e calcificações testiculares.

## Discussão

Os MCs são os tumores cardíacos primários mais comuns.^2^ No entanto, os casos de recidiva são muito raros.^7 , 8^ O CNC é um distúrbio genético incomum, passado hereditariamente de maneira autossômica dominante; caracterizado por múltiplos tumores benignos que acometem mais frequentemente o coração, a pele e o sistema endócrino; e alterações na pigmentação da pele, resultando em uma aparência manchada nas áreas afetadas. Manifesta-se aos 20 anos, em média, e sua prevalência permanece desconhecida.^5^

O diagnóstico é feito com pelo menos dois dos 12 critérios propostos por Stratakis ( Tabela 2 ) ou uma dessas alterações, além do acometimento de parente de primeiro grau ou mutação no gene da subunidade de tipo I reguladora da proteína quinase A [gene PRKAR1A].^5^

**Tabela 2:** – Principais critérios diagnósticos de acordo com Stratakis

1 - Lentiginose cutânea com distribuição típica (lábios, conjuntiva, mucosas)
2 - Mixoma (cutâneo e mucoso) ou mixoma cardíaco
3 - Achados de mixomatose mamária ou ressonância magnética sugestivos do diagnóstico
4 - Doença nodular primária pigmentada ou aumento paradoxal da excreção de glicocorticoides urinários após administração de dexametasona
5 - Acromegalia associada ao adenoma hipofisário que produz GH
6 - Tumor testicular de grandes células de Sertoli calcificadas ou presença de calcificações no ultrassom testicular
7 - Carcinoma tireoidiano ou presença de múltiplos nódulos hipoecóicos no ultrassom da tireoide em idade pré-púbere
8 - Schwannomas melanocíticos psammomatosos
9 - Nevo azul, nevo azul epitelioide múltiplo
10 - Adenomas ductais mamários múltiplos
11 - Osteocondromioma
**Critérios suplementares**
1 - Membro da família acometido
2 - Presença de mutações inativadoras do gene PRKAR1A
3 - Variantes ativadoras do gene PRKACA ou PRKACB

É importante abordar e monitorar casos individuais e familiares de mixomas recorrentes, pois, até o momento, foram descritas mais de 125 mutações do gene PRKAR1A. É o principal gene associado ao CNC.^9^ As mutações inativadoras do gene PRKAR1A são responsáveis pelas manifestações fenotípicas do CNC em mais de 70% dos casos.^6 , 9^

O gene PRKAR1A é um componente chave da via de sinalização celular do monofosfato cíclico de adenosina (cAMP) envolvido na tumorigênese. Portanto, essa patologia pode ser considerada uma forma de neoplasia endócrina múltipla com acometimento das glândulas adrenais, hipofisárias, tireoidianas e gônadas.^3 , 6 , 9^

A doença vascular cerebral pode ser a apresentação do MC.^10^ Além disso, as manifestações neurológicas têm apresentação típica em pacientes jovens com predominância do sexo masculino, sendo a principal apresentação clínica do nosso caso.

## Conclusão

O CNC é uma entidade rara, associada a múltiplas manifestações cutâneas e endocrinológicas, estando relacionado ao aparecimento e recorrência de mixomas. Deve-se suspeitar de CNC em qualquer paciente com MC recorrente. Em pacientes diagnosticados com CNC, deve-se seguir uma abordagem completa e multidisciplinar, tanto no paciente quanto em parentes próximos, que atendam a alguns critérios diagnósticos, pois podem ser portadores de mutações no gene PRKAR1A. O diagnóstico do complexo de Carney deve ser considerado quando ele atender aos critérios de diagnóstico, mesmo que exames genéticos não estejam disponíveis ou confirmados.
