# Supplementary material for: Mixoma Atrial Recorrente em Paciente com Complexo de Carney. Relato de Caso e Revisão de Literatura
Source: Arq Bras Cardiol. 2020 May 11;114(4 Suppl 1):31–3. [Article in Portuguese] doi: 10.36660/abc.20190405 (PMC8149110; doi:10.36660/abc.20190405)
Supplement: Translation

# Translation: Recurrent Atrial Myxoma in a Patient with Carney Complex. A Case Report and Literature Review

**Authors:** Laura A. Cervantes-Molina, David Ramírez-Cedillo, Italo D. Masini-Aguilera, Jaime G. López-Taylor, Michel Machuca-Hernández, Dulman O. Pineda-De Paz

**Affiliations:** 1 México Hospital Civil de Guadalajara Unidad Hospitalaria Fray Antonio Alcalde - Cardiovascular Surgery, Guadalajara, Jalisco - México

**Keywords:** Carney Complex/complications, Atrial Myxoma, Genetic Loci, PRKAR1A

## Introduction

Primary cardiac tumors are uncommon. They have an incidence between 0.0017% and 0.28% corresponding to 17 and 2,800 of primary heart tumors per 1 million autopsies.^1^ Cardiac Myxoma (CM) is a benign neoplasm and represents the most common one among primary cardiac tumors in adults.^2^

CMs have an annual incidence of 0.5–0.7 of surgically resected cases per one million; with the majority of cases showing sporadic appearance and less than 10%, a family inheritance pattern.^3^

The left atrium (LA) is the most common site of origin (75–80%), followed by the right atrium. Multiple CMs represents 5% of all CMs and only 50% of them have a bilateral origin.^4^

CMs have a wide range of manifestations, mostly obstructive symptoms, but they can also produce embolisms being the worst of their scenarios.^1^

There are 2 types of CM: 1. Simple, the most common one, representing 95% of all CMs. Its highest prevalence is at 56 years of age, with a risk of developing a second myxoma between 1 and 3%. 2. Autosomal dominant family forms, such as Carney Complex (CNC).^2^

These types of CM present an atypical anatomic distribution that is different from the LA.^5^ They appear at an average age of 25 years and tend to be multiple in 45% of cases, with a relapse rate between 15% and 22%.^1 , 4 , 6^

## Case Report

We report the case of a 22-year-old male patient with a previous history of right atrial CM resection at 12 years of age and resection of a cutaneous abdominal myxoma at 20 years of age; he was brought to the ER for generalized sudden onset paresthesia associated with right fascio-corporal hemiparesis and motor aphasia. On physical examination, acromegalic-like appearance, short neck, lentiginosis with grayish nevi on his lower lip and multiple café-au-lait spots on his face ( Figure 1-A ).

**Figure 1:** - Panel A. Patient’s image showing grayish nevi (arrow) and café-au-lait spots (circle). Panel B. Axial computed tomography (CT scan) of the brain showing intra-axial hypodense image in the left parieto-temporal region of 40 * 24 mm (asterisk) with mild/moderate surrounding edema. Panel C. Apical four-chamber transthoracic view showing a large left atrial mass (asterisk) and panel D showing its correlation with surgical view (arrow). Panel E. Macroscopic view of left atrial myxoma. Panel F. Right testicular ultrasound shows multiple calcifications.

Brain axial CT scan showed intra-axial hypodense image in the left parietal-temporal region of 40 x 24 mm in diameter ( Figure 1-B ). Then, echocardiography was performed, showing normal LVEF (64%) and mild LA dilatation. LA mobile mass (5.4 cm x 2.8 cm in diameter) of homogeneous appearance and regular contours adhered to the interatrial septum with prolapse to the left ventricle without no gradient of stenosis or signs of regurgitation. ( Figure 1-C )

CNC was suspected, due to an abnormal hormonal profile [secondary hyperthyroidism and hypercortisolism]. ( Table 1 )

**Table 1:** – Endocrine profile

**IGF-1**	**922 ng/ml (z. Score: +4.8)**
ACTH	**22.6 pmol/l**
PRL	3.42 ng/dl
Cortisol	33.01 mcg/dl
TSH	7.26 mcU/ml
T4l	2.34

Additional tests were performed: brain MRI, which showed in axial sequence T1 a hypointense area of 7 mm in diameter, corresponding to a pituitary microadenoma and testicular ultrasound, which revealed bilateral microlithiasis ( Figure 1-F ).

As recommended in international guidelines, he was taken to cardiac surgery for tumor resection and concomitant resection of the atrial septum, because this is absolutely mandatory during the resection of CM in CNC to avoid atrial myxoma recurrence. LA mass of soft and friable consistency, compatible with CM of 5.5 cm x 3 cm was found ( Figure 1-D and E ). Histological analysis, again, confirmed atrial myxoma.

The patient had a favorable postoperative outcome, without any complication, and progressive improvement of neurological symptoms. CNC diagnosis was established mainly due to the multiple cutaneous disorders, “recurrent” and “bilateral” CM, prior history of extra-cardiac myxoma, as well as endocrine disorders and testicular calcifications.

## Discussion

CMs are the most common primary cardiac tumors.^2^ However, cases of recurrence are very rare.^7 , 8^ CNC is an uncommon genetic disorder inherited in an autosomal dominant manner; characterized by multiple benign tumors most often affecting the heart, skin and the endocrine system; and abnormalities in skin coloring (pigment) resulting in a spotty appearance to the skin of affected areas. Its mean age of presentation is 20 years, and its prevalence remains unknown.^5^

Diagnosis is made with at least two of the 12 criteria proposed by Stratakis ( Table 2 ) or one of these abnormalities plus the affection of a first-degree relative or mutation in the gene of regulatory subunit type I protein kinase A [gene PRKAR1A].^5^

**Table 2:** – Major diagnostic criteria according to Stratakis

1 - Cutaneous lentiginosis with typical distribution (lips, conjunctiva, mucous membranes)
2 - Myxoma (cutaneous and mucosal) or cardiac myxoma
3 - Mammary myxomatosis or MRI findings suggestive of the diagnosis
4 - Pigmented primary nodular disease or paradoxical increase in the excretion of urinary glucocorticoids after dexamethasone administration
5 - Acromegaly associated with pituitary adenoma producing GH
6 - Testicular tumor of large calcified sertoli cells or presence of calcifications in testicular ultrasound
7 - Thyroid carcinoma or presence of multiple hypoechoic nodules in prepubertal thyroid ultrasound
8 - Psammomatous melanocytic schwannomas
9 - Blue nevus, multiple epithelioid blue nevus
10 - Multiple ductal mammary adenomas
11 - Osteochondromyoma
**Supplementary Criteria**
1 - Affected family member
2 - Presence of inactivating mutations of the PRKAR1A gene
3 - Activating variants of the PRKACA gene or PRKACB gene

It is important to approach and monitor both individual and family cases of recurrent myxomas as, to date, more than 125 mutations of gene-PRKAR1A have been described. It is the main gene associated with CNC.^9^ The inactivating mutations of gene PRKAR1A are responsible for the phenotypic manifestations of CNC in more than 70% of cases.^6 , 9^

The gene PRKAR1A is a key component of the cellular signaling pathway of cyclic adenosine monophosphate (cAMP) involved in tumorigenesis. Therefore, this pathology could be considered a form of multiple endocrine neoplasia with involvement of the adrenal, pituitary, thyroid glands and gonads.^3 , 6 , 9^

Cerebrovascular disease can be the presentation of CM.^10^ Besides, neurological manifestations have a typical presentation in young patients with predominance in males, being the main clinical presentation of our case.

## Conclusion

CNC is a rare entity that is associated with multiple cutaneous and endocrinological manifestations, and is related to the appearance and recurrence of myxomas. CNC must be suspected in any patient with recurrent CM. In patients diagnosed with CNC, a complete and multidisciplinary approach should be followed both on the patient and on close relatives meeting some diagnostic criteria, since they could be carriers of mutations of gene PRKAR1A. Diagnosis of Carney complex should be considered when it fulfills the diagnostic criteria even if the genetic test is not available or confirmed.
